# Care Needs and Symptoms Burden of Breast Cancer Patients in Jordan: A Cross-Sectional Study

**DOI:** 10.3390/ijerph191710787

**Published:** 2022-08-30

**Authors:** Omar Shamieh, Ghadeer Alarjeh, Houshen Li, Mahmoud Abu Naser, Fadi Abu Farsakh, Rashid Abdel-Razeq, Adib Edilbi, Ruba Al-Ani, Richard Harding, Ping Guo

**Affiliations:** 1 Department of Palliative Care, King Hussein Cancer Center, 202 Queen Rania Al Abdulla St., Amman 11941, Jordan; 2 Center for Palliative & Cancer Care in Conflict, King Hussein Cancer Center, Amman 11941, Jordan; 3 Faculty of Medicine, The University of Jordan, Amman 11942, Jordan; 4 Cicely Saunders Institute, Florence Nightingale Faculty of Nursing, Midwifery and Palliative Care, King’s College London, London SE5 9PJ, UK; 5 Department of Medicine, King Hussein Cancer Center, Amman 11941, Jordan; 6 School of Nursing & Midwifery, Institute of Clinical Sciences, College of Medical and Dental Sciences, University of Birmingham, Birmingham B15 2TT, UK

**Keywords:** breast cancer, neoplasm, palliative care, curative, symptoms

## Abstract

Breast cancer is the most common malignancy in Jordan. Rigorous needs assessment for breast cancer patients can prioritize both cancer care and palliative care to propose the appropriate services effectively. We conducted a cross-sectional study of breast cancer patients in a cancer center in Jordan. We assessed symptom burden, comorbidities, and performance using the Edmonton Symptom Assessment System (ESAS), the Charlson Comorbidity Index (CCI), and the Australia-modified Karnofsky performance scale (AKPS). Descriptive analysis and regression models to predict the highest symptom burden were used. A total of 233 participants were enrolled: curative vs. palliative intent groups (147 (63%) vs. 86 (37%) patients), respectively. Tiredness was the most reported symptom in 189 patients (81%), while nausea was the least in 61 patients (26.2%). A relationship between the AKPS score and total ESAS was seen (correlation coefficient of −0.487; *p* < 0.0001). The prevalence of anxiety (*p* = 0.014), lack of appetite (*p* = 0.002), poor well-being (*p* < 0.001), and sleep disorder (*p* = 0.035) was higher in the palliative care intent group than in the curative one. We identified unmet needs in breast cancer patients. Both groups showed a prevalence of distressing symptoms suggesting that even those with non-palliative intent have high needs and should receive integrated palliative care.

## 1. Introduction

Palliative care aims to reduce the suffering of cancer patients and families by controlling patients’ symptoms and improving their quality of life. Yet, pain and symptom management are still suboptimal, especially in low- and middle-income countries [1,2]. Palliative care development in Arab countries is evolving, with varying levels of action [3]. Access to palliative care services is limited to a portion of the population, and most services are still primarily offered to cancer patients [4].

Jordan is a middle-income Arab country in southwest Asia with limited resources and a young population structure [5]. Breast cancer is the most common malignancy and the third leading cause of cancer death [6]. Therefore, breast cancer poses a particularly challenging burden on the country’s healthcare system. Data on breast cancer pathology, clinical presentation, and treatment outcomes in the region are limited and mostly represent retrospective analyses. Recruitment to clinical trials continues to be challenging, and patients are notably under-presentable [7].

Patients with breast cancer often experience an accumulating symptom burden, deterioration of function, and other concerns inherent to a slowly progressive, life-limiting disease [8]. They have significant multi-dimensional physical, psychological, social, emotional, and spiritual needs that may not be met [9]. Palliative care focuses on comfort, optimizing functional independence and quality of life for as long as possible rather than disease-directed or life-prolonging treatment [10] and is increasingly thought of as a useful addition to standard breast cancer care [11]. However, palliative care in Jordan is still growing and has far to go. It is mainly delivered by the largest cancer center, King Hussein Cancer Center—KHCC, the Army Hospital, the private sector, and a nongovernmental home hospice agency [12].

A rigorous assessment of breast cancer patients’ needs can prioritize cancer care and palliative care to propose the appropriate services and allocate resources effectively [13,14]. However, the actual needs of breast cancer patients in Jordan have not been identified [6]. In this study, we aim to describe the care needs of breast cancer patients attending an ambulatory breast cancer clinic in a tertiary cancer center in Jordan, who the palliative care team has never evaluated. The objectives were to establish baseline prevalence and severity of symptoms for breast cancer patients in Jordan, define demographic and clinical predictors of higher symptom burden in the Jordanian breast cancer context, and inform local and national clinical service planning. 

## 2. Materials and Methods

### 2.1. Study Design

This is a cross-sectional study of a consecutive case series conducted in KHCC, a tertiary cancer center in Jordan.

KHCC is a nongovernmental, non-for-profit center founded in 1997 that currently treats approximately 60% of cancer occurrences in Jordan and provides cancer treatment to patients from several countries in the surrounding region [15]. The palliative care program in KHCC is growing rapidly to serve the needs of cancer patients and their families [12,16]. KHCC provides palliative care through interdisciplinary teams, including nurses, doctors, psychosocial counselors, social workers, pastoral care workers, and volunteers.

### 2.2. Population

We collected consecutive case data from adult breast cancer patients attending the ambulatory breast cancer clinic who received anti-cancer therapy at KHCC between July 2015 and June 2016. Potential participants were approached by a research assistant and asked if they would be interested in participating in this study. All participants gave written informed consent. Eligible patients were adults (age ≥ 18 years) with a breast cancer diagnosis, receiving care at KHCC during the study period, and who were able to read and respond to questions in Arabic. We excluded patients who were unable or unwilling to consent or had a significant psychiatric or cognitive decline that would interfere with participation. Local ethical approval was obtained from the Institution Review Boards at KHCC (reference no.14 KHCC 44).

### 2.3. Data Collection

Demographic data (including patient age, gender, marital status, occupation) and clinical information (stage of breast cancer at diagnosis) were collected. Information about treatment received (chemotherapy, radiotherapy, immunotherapy, surgery, targeted therapy) and therapy goals (curative intent vs. palliative intent as determined at the point of care) were also collected from the patient’s medical records and confirmed with the primary oncologist.

### 2.4. Assessments

ESAS: The Edmonton Symptom Assessment Scale [17] is a validated scale measuring the intensity of nine common symptoms experienced by cancer patients. Participants were asked to rate their symptoms from 0 to 10, with 0 signifying no distress and 10 signifying the worst distress. The symptoms were pain, tiredness, nausea, depression, anxiety, drowsiness, appetite, well-being, shortness of breath, and sleep. The total sum of ESAS symptoms range from 0 to 100 [18].

CCI: Comorbidity was assessed using the Charlson Comorbidity Index, a valid tool used to address multiple comorbidities by creating a weighted total score based on the presence of various conditions, thereby generating the patient’s risk of death [19]. The index contained 19 comorbid conditions. A CCI score of 0–1 indicates low comorbidity, and a score of 2 or higher is considered high comorbidity [19]. Comorbid conditions were recorded if present in the medical record and confirmed with the participant. 

AKPS: Performance status was measured and collected by clinic nurses and double-checked by the research assistant with the Australia-modified Karnofsky Performance Scale (AKPS) [20]. This scale is a blend of the original golden standard scale, the Karnofsky Performance Scale (KPS), and the community-based care-focused scale; the Thorne modified KPS was formulated to suit all care settings. This scale is categorized according to patients’ performance scale, which ranges from 0, the patient is dead, to 100, the patient is considered normal with no complaints and no evidence of disease [20].

### 2.5. Statistical Analysis

Symptoms were described and cross-tabulated with age, the intent of therapy, CCI, and function measured by AKPS. Correlations were checked between key parameters. A regression model was constructed to seek predictors of the highest symptom burden to target future interventions. 

The responses on the ESAS (0 = absent to 10 = extremely severe) for each symptom were grouped into four categories according to the responses: absent (0), mild (1, 2, 3), moderate (4, 5, 6), and severe (7, 8, 9, 10) [21]. We compared symptom severity between the curative intent group and the palliative intent group (curative intent group treatment aimed to achieve complete remission and prevent a recurrence, while the palliative intent group focused on no cure but simply decreasing tumor burden and prolonging life expectancy) [22] by Chi-Square tests. In line with previous validation studies, both choices of ‘moderate’ and ‘severe’ were treated as clinically significant options [23].

The bivariate relationship between the total ESAS and AKPS was plotted. Linear regression was used to predict AKPS from ESAS. The strength of the association between total sum of ESAS symptoms and the AKPS score was investigated using Kendall’s Tau-b correlation coefficient as the best measure of association between two ordered categorical variables. We used R Version 4.0.3 (R Foundation for Statistical Computing, Vienna, Austria) for the statistical analyses [24].

## 3. Results

A total of 233 participants were enrolled and assigned to either the curative intent group (n = 147) or palliative intent group (n = 86), of whom one was male, and 232 were female participants (Table 1). Most participants were Jordanian and Muslims. The median age in both groups was 48 years. Marital status, employment status, and educational level did not differ between groups at baseline. The results showed that the AKPS was significantly lower in the palliative intent group, with a median of 80% (*p* < 0.0001). Patients with advanced cancer (stage III and IV) represented a significantly higher percentage of the palliative intent group when compared to the curative intent group (81.4% vs. 42.8%; *p* < 0.0001). While the severity of the total symptoms was significantly (*p* = 0.006) higher in the palliative intent group, the median (range) of total ESAS for this group was 35 (1–86) compared to 20 (0–65) in the curative intent group. Moreover, patients with palliative intent therapy had a significantly (*p* < 0.0001) higher median (range) of their CCI 7.5 (2–12) compared to 4 (2–9) for patients with curative intent therapy, as shown in Table 1.

### 3.1. Symptom Burden

Out of 233 patients, 228 (97.9%) reported some discomfort on the ESAS, while only 5 patients reported no discomfort. A total of 160 patients (68.7%) had pain. Nearly 99 (42.5%) patients had moderate pain, and 54 (23.1%) patients had severe pain. Tiredness was the most reported symptom among patients. Out of 189 patients who had tiredness, 55 patients reported (29.1%) mild fatigue, 72 patients reported moderate fatigue (38.1%), and 62 patients experienced severe fatigue (32.8%). The least prevalent symptom among participants was nausea, which was only reported by 61 out of 233 patients (26.2%), as shown in Table 2.

### 3.2. Correlation between AKPS and Total ESAS Score

**Figure 1:**
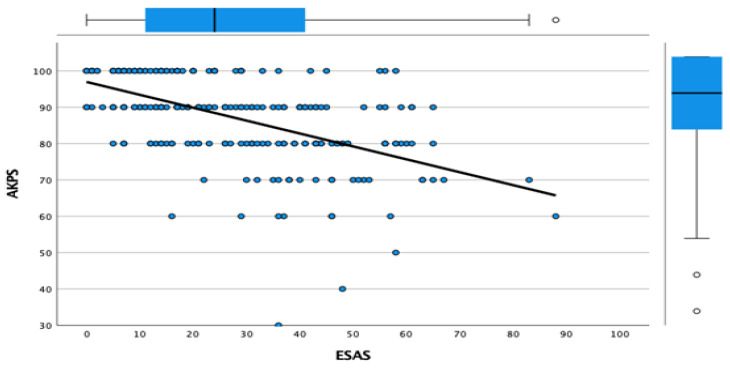
Liner regression of physical function and symptom severity. AKPS: Australia-modified Karnofsky performance scale, ESAS: Edmonton Symptom Assessment Scale.

The mean AKPS declined as the symptoms got more severe, with a steeper exponential decline when patients’ total ESAS was over 60. No individual was observed to have an AKPS score of less than 60%. The linear regression to predict AKPS from each ESAS group category is shown in Figure 1, and the values are given in Table 2. A relationship is demonstrated (*p*-value for trend <0.0001), although this is not strong (adjusted R^2^ = 0.300). Predicted AKPS from the total ESAS gave the following values: ESAS 0–20, predicted KPS 90–100%; ESAS 20–40, predicted AKPS 80–90%; ESAS 40–80, predicted KPS 70–80%; and ESAS > 80, predicted AKPS 60%. Using Kendall’s Tau-b correlation coefficient, a relationship between the AKPS score and total ESAS was seen (correlation coefficient of −0.487; a negative value reflecting the inverse relationship; *p* < 0.0001). A moderate strength of agreement was demonstrated between the observed and predicted AKPS scores.

### 3.3. Curative Intent vs. Palliative Intent

Regarding the goal of anti-cancer therapy, 147 (63.1%) received treatment with curative intent, and 86 (36.9%) received treatment with palliative intent. The prevalence of anxiety (*p* = 0.014), lack of appetite (*p* = 0.002), poor well-being (*p* < 0.001), and sleep disorder (*p* = 0.035) was higher in the palliative care intent group than in the curative care intent group (Table 2). Poor wellbeing (Cramer’s V = 0.270) had a very strong relationship with the goal of palliative intent, while lack of appetite (Cramer’s V = 0.214), anxiety (Cramer’s V = 0.182), and sleep disorder (Cramer’s V = 0.164) had a strong relationship with the goal of palliative care where Cramer’s V: >0.25 = Very strong; >0.15 = Strong; >0.10 = Moderate; >0.05 = Weak; >0 = No or very weak [25].

**Figure 2:**
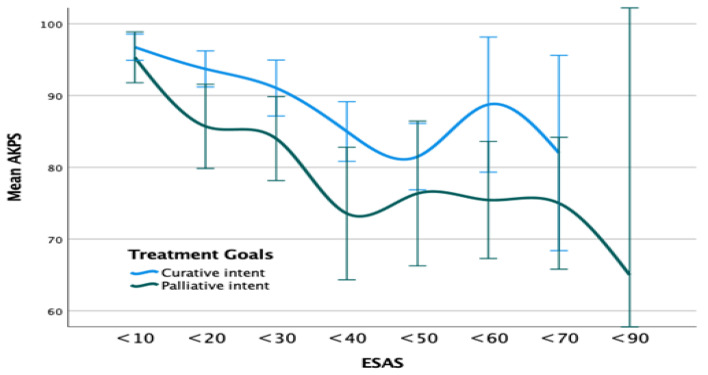
Mean physical function and symptom severity by treatment goals. AKPS: Australia-modified Karnofsky performance scale, ESAS: Edmonton Symptom Assessment Scale.

We explored potential differences in symptom burden by physical function status between patients with curative intent and those with palliative intent. The change in physical function by AKPS was almost identical across the two groups at each severity of symptom burden, with identical mean AKPS scores (Figure 2). Patients with curative intent had better mean AKPS in all ESAS groups. Both groups had a steeper decrease between total ESAS rated 10 and 40, while the palliative intent group had a moderate decrease from total sum of ESAS rated 50 to 90.

## 4. Discussion

This study represents the largest regional cancer hospital in Jordan. Our findings identified the unmet needs of breast cancer patients and the factors determining the groups with the highest likelihood of burdensome symptoms, potentially influencing clinical decisions and local policymaking.

Anxiety, lack of appetite, poor well-being, and sleep disorder seem to be the most statistically significant symptoms that may affect breast cancer patients and interfere with their quality of life, especially for those whose goal of care is palliative care. The findings probably match the patterns of breast cancer care seen in other resource-limited Arab countries. Models of cancer care differ between health systems, but the findings reasonably represent a cohort of people with unmet needs who need accessible palliative care provision. In another cultural context, a Malaysian study found that most patients with breast cancer suffered from unmet needs of psychological issues such as uncertainty regarding their future (78.6%) and feeling depressed (65.0%), followed by physical symptoms including feeling unwell (58.1%), fatigue (57.3%), and pain (55.6%) [26].

Patients with breast cancer have complex needs and limited referrals to palliative care services as the trajectory of the disease declines [27]. In this preliminary exploration, we have evaluated symptoms and function; where fatigue was the most reported symptom, the total ESAS class and AKPS score showed an inverse relationship, and the prevalence of ESAS symptoms was higher in the palliative care intent group in a cohort of breast cancer patients at KHCC, Jordan.

We recorded a statistically and clinically significant severity of tiredness, well-being, and pain in people with breast cancer, which is similar to Costa and colleagues’ study (2017) in that the majority of breast cancer patients involved in the study complained of pain that was inversely correlated with the patient’s quality of life [28]. Moreover, fatigue was a persistent issue for female breast cancer patients. It increased significantly with diagnosis and lasted for more than 6 months before showing its gradual and slow decline in intensity over time, which may last for several years after treatment [29].

Unlike pain or shortness of breath, for which a meaningful improvement has been defined, assessing a meaningful improvement in tiredness relies on the threshold of discrimination for changes in health-related quality of life; for chronic diseases this appears to be approximately half an SD [30]. Identifying such patterns and finding more effective and standardized ways to respond to such changes might help to improve the quality of life in people with breast cancer. Similarly, we recorded nearly identical patterns of symptom burden among both goals of care groups of patients. Hence, integration and collaboration between oncology and palliative care are recommended to improve patients’ quality of life and outcomes during their entire disease journey, especially in their advanced stages of illness [27].

The use of both the AKPS score and ESAS in studies with breast cancer may add clarification to the description of the population. Using both scales even when AKPS is over 60 may provide the clinician with additional useful information, and for breast cancer patients with poor ESAS scores, routine application of the AKPS in clinical practice may provide a better prompt for assessing palliative care needs, given the wide range of performance scales encompassed within this category. This is similar to the Mather et al. study. However, different outcome measures (the Palliative Care Problem Severity Scale, AKPS, and phases of illness) were used to give a clearer insight into palliative patients’ clinical situations and needs [31].

The use of patient-centered outcome measures (PCOMs) in a palliative care setting is important for identifying patients’ needs and improving and demonstrating quality of care. However, the implementation of PCOMs is challenging and needs regular feedback, collaboration, and facilitators to support its routine use [32].

Usually, in the current practice, breast cancer patients are not being assessed for symptoms or functionality using ESAS and AKPS unless the palliative care team at KHCC follows them. Many symptoms and complaints may be missed because of the lack of use of well-developed PCOMs.

A well-developed PCOM can improve and facilitate communication and goals of care discussions between patients and healthcare providers, which is one of the most important core roles of the palliative care team [32].

According to the study results, both groups of breast cancer patients (curative and palliative care intent) showed a prevalence of distressing symptoms such as fatigue and pain that may interfere with their functionality and quality of life. Hence, assessing and addressing patients’ functionality, needs, and symptoms is crucial to properly manage and improve patient care and quality of life [32].

Addressing breast cancer patients’ needs using outcome measures will help in symptom recognition, palliative referral, and improving patients’ quality of care [32]. Furthermore, the study results support the need for outcome measurer tools that may inform cancer care policy and guidelines in Jordan.

This study is unique and has several strengths. First, we recruited patients from KHCC, where most cancer patients were treated in Jordan. This enabled us to explore the clinical feature of breast cancer patients in the country. Second, symptoms were collected by a patient-reported outcome scale, thus providing a high-quality prospectively collected data source. This feature enabled us to explore symptoms in a population often deemed too difficult or costly to access for such research.

There are some inherent limitations in our study. First, we described the symptom burden among patients treated for breast cancer in one large cancer center. This is a geographically limited sample. Further study is needed in other centers in the country to test these findings. Second, this was a non-community cohort of patients treated in the hospital. As such, it was not necessarily representative of patients in the community or those who were discharged and died at home amid recent treatment, as is sometimes the case for patients with breast cancer.

Further study is needed on breast cancer care of community or home-based patients who are not referred to hospital services. Third, the ESAS assesses just ten symptoms. Other prominent or bothersome symptoms might be experienced differently by patients with varying life-limiting illnesses, which are not assessed by these questions. Moreover, the socio-economic status data of enrolled breast cancer patients were not collected. Other studies reported that the socio-economic status of such a group of patients was associated with higher levels of inflammation and poor cancer-related outcomes [33]. For example, Choi et al. found that breast cancer patients with poor socio-economic conditions suffered from higher pain levels than patients with less distressful conditions and concluded that pain management should be tailored to patients’ social determinants [34]. Furthermore, recent data might be needed, although not necessarily different from our findings.

## 5. Conclusions

Our findings identified the unmet needs of breast cancer patients and the factors determining the group with the highest burdensome symptoms. Both groups of breast cancer patients (curative and palliative care intent) showed a prevalence of distressing symptoms such as pain, fatigue, anxiety, poor appetite, and poor well-being that interfere with their quality of life, suggesting even those with non-palliative intent still have high needs and should get some integrated palliative care. Identifying these factors and symptoms will potentially influence clinical decisions and local policymaking. We suggest developing a proper protocol and guidelines with tailored measures for all cancer patients regardless of their cancer type, stage, and therapy intent. These protocols will help healthcare providers know which symptoms and needs should be addressed before and after proper treatment. This guidance should be used in all hospitals in Jordan and can be adapted in other middle- to low-income countries.

## Figures and Tables

**Table 1:** Demographic and clinical characteristics of patients with breast cancer (n = 233).

	All Patients (n = 233) No. (%)	Curative Intent (n = 147) No. (%)	Palliative Intent (n = 86) No. (%)	*p* Value
**Median age** at diagnosis in years (range)	48 (24–78)	48 (28–77)	48 (24–78)	
**Gender**				1.000
Female	232 (99.6%)	146 (99.3%)	86 (100%)	
Male	1 (0.4%)	1 (0.7%)	0	
**Nationality**				0.540
Jordanian	222 (95.3%)	141 (95.9%)	81 (94.2%)	
Non-Jordanian	11 (4.7%)	6 (4.1%)	5 (5.8%)	
**Marital status**				0.850
Single	13 (5.6%)	7 (4.8%)	6 (7.0%)	
Married	194 (83.3%)	124 (84.4%)	70 (81.4%)	
Divorced	4 (1.7%)	3 (2.0%)	1 (1.2%)	
Widowed	21 (9.0%)	12 (8.2%)	9 (10.5%)	
Separated	1 (0.4%)	1 (0.7%)	0	
**Religion**				0.824
Muslim	210 (90.1%)	132 (89.8%)	78 (90.7%)	
Christian	23 (9.9%)	15 (10.2%)	8 (9.3%)	
**Employment status**				0.454
Still working	43 (18.5%)	31 (21.1%)	12 (14.0%)	
Overtime work	1 (0.4%)	1 (0.7%)	0	
Retired	32 (13.7%)	21 (14.3%)	11 (12.8%)	
Housewife	122 (52.4%)	76 (51.7%)	46 (53.5%)	
Do not work	32 (13.7%)	16 (10.9%)	16 (18.6%)	
Others	3 (1.3%)	2 (1.4%)	1 (1.2%)	
**Education level**				0.638
Primary education	25 (10.7%)	16 (10.9%)	9 (10.5%)	
Secondary education	77 (33.0%)	45 (30.6%)	32 (37.2%)	
Diploma	53 (22.7%)	35 (23.8%)	18 (20.9%)	
Bachelor’s degree	66 (28.3%)	45 (30.6%)	21 (24.4%)	
Postgraduate degree	12 (5.2%)	6 (4.1%)	6 (7.0%)	
**Stage at diagnosis**				<0.0001
I	19 (8.2%)	16 (10.9%)	3 (3.5%)	
II	81 (34.8%)	68 (46.3%)	13 (15.1%)	
III	84 (36.1%)	60 (40.8%)	24 (27.9%)	
IV	49 (21.0%)	3 (2.0%)	46 (53.5%)	
**Treatment received**				
Radiotherapy	115	64 (43.5%)	51 (59.3%)	0.020
Chemotherapy	214	135 (91.8%)	79 (91.9%)	0.995
Immunotherapy	0	0 (0%)	0 (0%)	NA
Surgery	168	124 (84.4%)	44 (51.2%)	0.000
Targeted therapy	150	89 (60.5%)	61 (70.9%)	0.110
Median AKPS (range)	90 (30–100)	90 (60–100)	80 (30–100)	<0.0001
Median ESAS total score (range)	24 (0–88)	20 (0–65)	35 (1–86)	0.006
Median CCI total score (range)	5 (2–12)	4 (2–9)	7.5 (2–12)	<0.0001

Note: NA: Not applicable, AKPS: Australia-modified Karnofsky performance scale, ESAS: Edmonton Symptom Assessment Scale, CCI: Charlson Comorbidity Index.

**Table 2:** Reported symptoms on the ESAS.

Symptoms on ESAS	All Patients (n = 233) No. (%)	Curative Intent (n = 147) No. (%)	Palliative Intent (n = 86) No. (%)	*p* Value
**Pain**				0.398
Absent	73 (31.3%)	45 (30.6%)	28 (32.6%)	
Mild	55 (23.6%)	39 (26.5%)	16 (18.6%)	
Moderate	68 (29.2%)	41 (27.4%)	27 (31.4%)	
Severe	37 (15.9%)	22 (15.0%)	15 (17.4%)	
**Tiredness**				0.153
Absent	44 (18.9%)	28 (19.0%)	16 (18.6%)	
Mild	55 (23.6%)	40 (27.2%)	15 (17.4%)	
Moderate	72 (30.9%)	44 (29.9%)	28 (32.6%)	
Severe	62 (26.6%)	35 (23.8%)	27 (31.4%)	
**Nausea**				0.384
Absent	172 (73.8%)	112 (76.2%)	60 (69.8%)	
Mild	24 (10.3%)	14 (9.5%)	10 (11.6%)	
Moderate	22 (9.4%)	14 (9.5%)	8 (9.3%)	
Severe	15 (6.4%)	7 (4.8%)	8 (9.3%)	
**Depression**				0.244
Absent	125 (53.6%)	83 (56.5%)	42 (48.8%)	
Mild	42 (18%)	28 (19.0%)	14 (16.3%)	
Moderate	32 (13.7%)	18 (12.2%)	14(16.3%)	
Severe	34 (14.6%)	18 (12.2%)	16 (18.6%)	
**Anxiety**				0.014
Absent	86 (36.9%)	58 (39.5%)	28 (32.6%)	
Mild	48 (20.6%)	29 (19.7%)	19 (22.1%)	
Moderate	51 (21.9%)	37 (25.2%)	14 (16.3%)	
Severe	48 (20.6%)	23 (15.6%)	25 (29.1%)	
**Drowsiness**				0.165
Absent	93 (39.9%)	63 (42.9%)	30 (34.9%)	
Mild	52 (22.3%)	32 (21.8%)	20 (23.3%)	
Moderate	52 (22.3%)	34 (23.1%)	18 (20.9%)	
Severe	36 (15.5%)	18 (12.2%)	18 (20.9%)	
**Appetite**				0.002
Absent	116 (49.8%)	83 (56.5%)	33 (38.4%)	
Mild	43 (18.5%)	28 (19.0%)	15 (17.4%)	
Moderate	51 (21.9%)	24 (16.3%)	27 (31.4%)	
Severe	23 (9.9%)	12 (8.2%)	11 (12.8%)	
**Well-being**				<0.001
Absent	69 (29.6%)	50 (34.0%)	19 (22.1%)	
Mild	53 (22.7%)	38 (25.9%)	15 (17.4%)	
Moderate	77 (33%)	48 (32.7%)	29 (33.7%)	
Severe	34 (14.6%)	11 (7.5%)	23 (26.7%)	
**SOB**				0.450
Absent	151 (64.8%)	99 (67.3%)	52 (60.5%)	
Mild	32 (13.7%)	20 (13.6%)	12 (14.0%)	
Moderate	34 (14.6%)	18 (12.2%)	16 (18.6%)	
Severe	16 (6.9%)	10 (6.8%)	6 (7.0%)	
**Sleep**				0.035
Absent	92 (39.5%)	59 (40.1%)	33 (38.4%)	
Mild	51 (21.9%)	38 (25.9%)	13 (15.1%)	
Moderate	45 (19.3%)	23 (15.6%)	22 (25.6%)	
Severe	45 (19.3%)	27 (18.4%)	18 (20.9%)	

Note: ESAS: Edmonton Symptom Assessment Scale, SOB: Shortness of breath.
